# Adaptive MDA-Net: Degradation-Aware Retinal Fundus Image Enhancement with Frequency-Domain and Illumination Guidance for Clinical Screening

**DOI:** 10.3390/diagnostics16162555

**Published:** 2026-08-13

**Authors:** Mohamed Loey, M. A. El-Mowafy, Mohamed A. Hemdan, Ramez Yousri, Heba M. Khalil

**Affiliations:** 1 Electrical Engineering and Computer Science, College of Engineering (CoE), A’Sharqiyah University (ASU), Ibra 400, Oman; 2 Department of Computer Science, Faculty of Computers and Artificial Intelligence, Benha University, Benha 13518, Egypt; heba.khalil@fci.bu.edu.eg; 3 Computer Science Department, Electronic Research Institute, Cairo 12622, Egypt; mohamedelmowafy@eri.sci.eg; 4 Department of Computer Science, Faculty of Computer and Information Systems, ECU, Nasr City 11765, Egypt or m.ahmed@ecu.edu.eg (M.A.H.); ramez.yousri@ecu.edu.eg (R.Y.)

**Keywords:** retinal fundus image enhancement, degradation-aware deep learning, image restoration, diabetic retinopathy screening, frequency-domain loss

## Abstract

**Background/Objective:** Retinal fundus image quality directly affects ophthalmic interpretation and automated analysis. Existing multi-degradation methods provide adaptive restoration but offer limited spatial degradation encoding and frequency-level supervision for severely degraded images. This study aims to improve restoration fidelity while preserving retinal structures required by downstream clinical-analysis models. **Methods:** Adaptive MDA-Net combines a CNN degradation encoder, coupled synthetic degradation, latent-conditioned attention, a supervised illumination-guidance branch, and a seven-term spatial- and frequency-domain objective. The model was evaluated on held-out EyeQ data and with fixed downstream models on DRIVE and REFUGE. **Results:** Across five runs, the model achieved 35.86 ± 0.12 dB PSNR and 0.9732 ± 0.0010 SSIM, improvements of 0.62 dB and 0.0064 over RGB-GAP. Downstream evaluation achieved a Dice score of 0.7601 on DRIVE and an mDice score of 0.7917 on REFUGE. **Conclusions:** Degradation-aware conditioning improves restoration fidelity and recovers part of the downstream performance lost to image degradation. Validation remains limited to image-level EyeQ splits and synthetic supervision.

## 1. Introduction

Retinal fundus photography supports screening and monitoring of diabetic retinopathy, glaucoma, and age-related macular degeneration [1,2]. Image quality is especially important for automated vessel, lesion, and disease-analysis systems, which depend on preserved anatomical boundaries and reliable colour information [3,4]. In routine acquisition, mixed exposure, illumination, blur, noise, and colour artefacts can obscure these features and reduce diagnostic and algorithmic reliability [5,6,7].

MDA-Net introduced multi-degradation-aware enhancement through adaptive filtering, but two gaps remain relevant for severe mixed degradation: its degradation representation does not explicitly preserve spatial and textural cues before global aggregation, and its restoration objective does not directly constrain frequency-domain fidelity [8]. These limitations motivate a model that learns an interpretable degradation signal, uses it to condition feature restoration, and separates illumination guidance from structural refinement.

We therefore propose Adaptive MDA-Net. The method replaces direct RGB-level degradation statistics with a lightweight CNN encoder supervised by the known parameters of a coupled degradation simulator. The resulting latent vector modulates channel and spatial attention, while a separately supervised illumination-guidance branch supports correction before structural refinement. Pixel, perceptual, edge, frequency, degradation-parameter, illumination, and smoothness objectives jointly train the framework.

The main contributions are:

1. A CNN degradation encoder with auxiliary parameter-regression and latent-dimension validation;
2. A reproducible coupled simulator whose synthetic distributions are compared with EyeQ Usable and Reject images;
3. An illumination-guided encoder–decoder with degradation-conditioned attention and explicit spatial/frequency supervision;
4. Evaluation using repeated restoration runs, no-reference quality measures, computational profiling, and fixed downstream models on DRIVE and REFUGE.

The remainder of the paper reviews related enhancement and conditioning methods in Section 2, presents Adaptive MDA-Net in Section 3, describes the experimental protocol in Section 4, reports results in Section 5, and concludes with discussion and future work in Section 6 and Section 7.

## 2. Related Work

### 2.1. Retinal Image Enhancement

Traditional retinal enhancement uses histogram adjustment, filtering, morphological processing, and illumination correction. Histogram equalisation and CLAHE improve local contrast but can amplify noise or alter brightness, whereas Wiener, median, multiscale, and morphological filters depend strongly on hand-selected parameters and may suppress fine vessels [9,10,11]. Retinex and homomorphic approaches address spatial illumination variation, but fixed decomposition assumptions can limit performance across devices and acquisition conditions [12]. These limitations motivate learned restoration models that adapt to image content and degradation severity.

Supervised CNN and Retinex-based networks learn mappings from degraded to high-quality images and can jointly address contrast, illumination, and noise [13,14,15,16]. Unpaired and self-supervised methods reduce reliance on registered image pairs through adversarial, transport, teacher–student, or consistency objectives [17,18,19,20]. However, generative enhancement can introduce unsupported structures or style changes, which is a safety concern in medical imaging [21,22]. This motivates deterministic restoration with explicit structural constraints.

### 2.2. Degradation Modelling

Paired clinical restoration data are scarce, so synthetic degradation is often used for training. Sequential simulators are reproducible but may not represent the co-occurrence of optical, sensor, illumination, exposure, and colour artefacts [8,18]. Coupled simulation instead samples interacting degradation factors and permits direct supervision of their known parameters. The present work follows this strategy and evaluates overlap between synthetic and real EyeQ quality distributions; it does not claim to reproduce the complete physical acquisition process.

### 2.3. Attention and Degradation Conditioning

Channel attention recalibrates feature channels, while spatial attention localises informative or degraded regions [23,24]. Dynamic Filter Networks adapt convolutional kernels to the input [25]; MDA-Net uses this principle for retinal enhancement [8]. Feature-level conditioning provides a lighter alternative by modulating intermediate activations using a learned latent representation, as in FiLM and conditional normalisation [26,27]. Adaptive MDA-Net combines feature-level modulation with channel and spatial attention so that a shared restoration backbone can respond to different degradation profiles.

### 2.4. Synthesis and Research Gap

Existing work establishes the value of learned enhancement, synthetic degradation, and adaptive processing, but rarely combines a spatially informed degradation encoder, explicit degradation-parameter supervision, supervised illumination guidance, and frequency-domain restoration in one retinal model. Adaptive MDA-Net addresses this gap while retaining deterministic reconstruction and evaluating whether enhancement benefits fixed downstream clinical-analysis networks.

## 3. Methods

### 3.1. Problem Formulation

Retinal fundus image quality is degraded by a heterogeneous set of acquisition artefacts, including uneven illumination, optical blur, sensor noise, and device-dependent colour imbalance, all of which can vary simultaneously and interdependently across clinical settings. Conventional enhancement frameworks typically apply uniform processing to all inputs, thereby failing to adapt to the specific degradation profile of each image.

To address this limitation, we propose Adaptive MDA-Net, a degradation-aware architecture designed for robust multi-condition retinal image enhancement. Rather than applying fixed filters, our approach explicitly models degradation characteristics and dynamically adapts restoration behaviour at the feature level. The framework integrates three tightly coupled components: (i) a degradation-aware latent representation module, (ii) an adaptive encoder–decoder backbone with Dynamic Residual Blocks, and (iii) an illumination-guided multi-stage refinement pipeline. This integrated design enables feature-level modulation rather than filter-level adaptation. The resulting deterministic restoration strategy is evaluated for structural preservation and downstream task performance, but clinical deployment is not claimed by the present study.

### 3.2. Data Preparation and Synthetic Degradation Modelling

To overcome the scarcity of paired clinical data, we design a synthetic degradation pipeline that generates representative degraded–clean image pairs from high-quality fundus images serving as ground truth. All images are resized to a fixed spatial resolution of 256 × 256 pixels and normalised to a symmetric intensity range [−1, 1], which stabilises training and accelerates gradient convergence.

We introduce an Adaptive Degradation Simulator that departs from the independent, sequential degradation models adopted in prior work. Instead of applying each artefact in isolation, the simulator implements a coupled, blended pipeline in which degradation components interact, providing a controlled approximation of selected, interdependent acquisition artefacts. Five degradation types are modelled:

- **Illumination Non-Uniformity:** Simulated via spatially varying attenuation masks and vignetting effects, combined with gamma correction, to mimic uneven lighting arising from acquisition geometry.
- **Exposure Variations:** Both underexposed and overexposed conditions are modelled through brightness scaling and gamma correction.
- **Optical Blur:** Multi-level Gaussian filtering simulates defocus and minor motion blur commonly encountered in clinical acquisitions.
- **Sensor Noise:** Additive Gaussian noise emulates stochastic sensor artefacts prevalent in low-light acquisition conditions.
- **Colour Perturbations:** Random channel-wise RGB scaling simulates device-dependent colour imbalance and acquisition inconsistencies, a component absent from the original MDA-Net.

Low-, mid-, and high-severity levels are selected with equal probability. Within a selected level, under-exposure and over-exposure are selected with probability 0.5. Every degradation component is applied to each synthetic sample, with continuous parameter draws from the level-specific intervals in Table 1. Per-image randomness denotes a new draw each time an image is presented, per-channel randomness denotes independent RGB scale factors, and per-level randomness denotes sampling from the selected severity interval.

Let (x, y) ∈ [−1, 1] denote normalised spatial coordinates. The simulator is defined by

(1) V(x, y) = clip(1 − s_v(x^2^ + y^2^), 0.30, 1.00),

(2) I_e = clip(β(I_h ⊙ V)^γ^, 0, 1),

(3) I_n = clip((I_e + ε) ⊙ c, 0, 1), ε~N(0, σ_n^2^),

(4) I_l = 2 clip(αI_n + (1 − α)G_r(I_h), 0, 1) − 1,

where V is the vignette mask, sv is vignette strength, γ and β control gamma and brightness, c = (sR, sG, sB) contains independent channel scales, Gr is Gaussian blur with radius r, σn is noise standard deviation, and α is the blend coefficient.

Adaptive Degradation Simulation Algorithm: Given a clean image and a seeded randomnumber generator: (1) select a severity level uniformly; (2) select under or over-exposure with probability 0.5; (3) sample gamma and brightness from the corresponding intervals; (4) sample blur radius, noise standard deviation, vignette strength, blend coefficient, and independent RGB scales; (5) construct V (x, y); (6) compute Ie; (7) add Gaussian noise and channel scaling to obtain In; (8) blend In with Gr(Ih) and map the result to [−1, 1]; and (9) return the degraded image, normalised nine-parameter target, attenuation mask, and unnormalised parameter dictionary.

For every generated training pair, the simulator also retains the exact normalised degradation parameters used to construct the degraded input. These auxiliary targets are collected in a vector θ = [γ, β, σb, σn, v, sR, sG, sB, α], where σb and σn denote blur and noise severity, v denotes vignette strength, and sR, sG, and sB denote the channel-wise colour scales. The spatial attenuation mask M_v is retained separately as the synthetic target for the illumination-guidance branch. These quantities provide training supervision only for synthetically degraded images and do not assume the availability of degradation labels or physical illumination maps for real clinical images.

### 3.3. Adaptive MDA-Net Architecture

**Figure 1:**
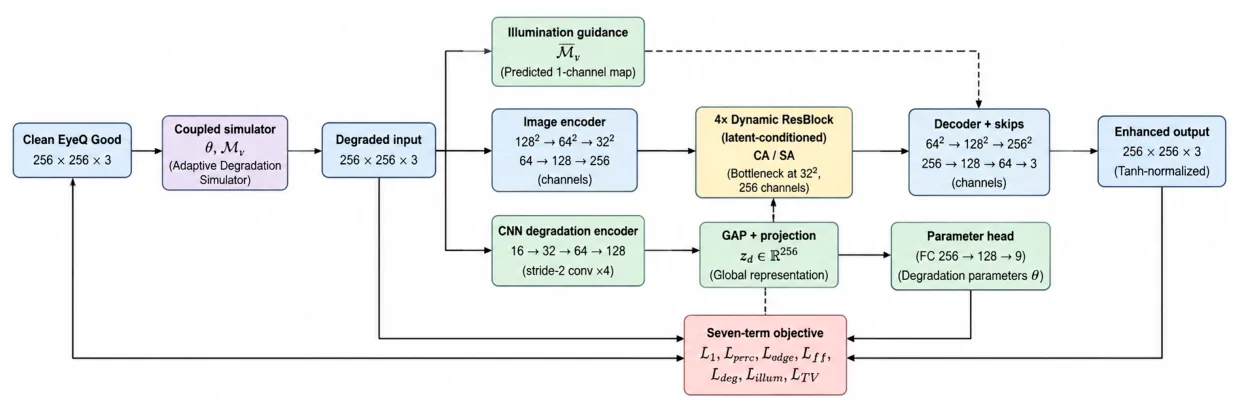
Adaptive MDA-Net training and inference flow. The degradation and illumination branches condition the encoder–decoder, while synthetic parameters, attenuation masks, and clean targets provide the seven training signals.

The proposed Adaptive MDA-Net comprises three main components: a degradation-aware latent representation module (Section 3.3.1), an adaptive encoder–decoder backbone (Section 3.3.2), and an illumination-guided multi-stage refinement pipeline (Section 3.3.3). A comprehensive overview of the full framework is illustrated in Figure 1.

#### 3.3.1. Degradation-Aware Latent Representation

**Figure 2:**
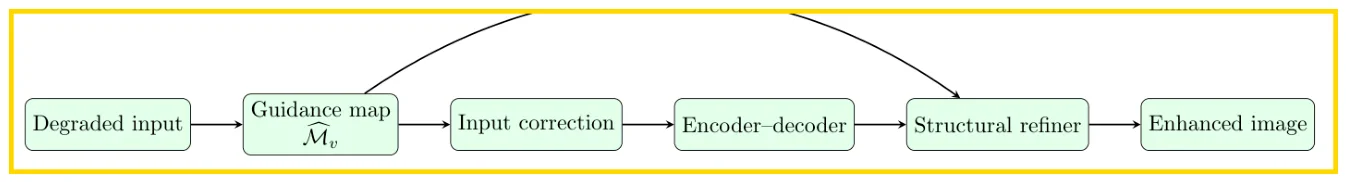
Illumination-guided refinement. The predicted map conditions both the pre-encoder correction and the post-decoder structural refiner.

The degradation encoder preserves spatial and textural evidence before Global Average Pooling and projects the resulting features to a 256-dimensional latent vector zd. This vector is broadcast to the Dynamic Residual Blocks, where it modulates channel and spatial attention responses as shown in Figure 2.

A 256 → 128 → 9 regression head predicts the normalised simulator parameters θ and supplies the auxiliary loss described in Section 3.4. This supervision encourages degradation sensitivity but does not establish complete separation between image content and degradation.

#### 3.3.2. Adaptive Encoder–Decoder Backbone

The backbone follows the dimensions summarised in Table 2. Three stride-2 encoder stages produce 64, 128, and 256 channels at 128^2^, 64^2^, and 32^2^ resolution. Four Dynamic Residual Blocks process the bottleneck features, and three transposed convolutions reconstruct the output using additive encoder skip connections.

Each Dynamic Residual Block applies two convolution–normalisation layers, latent-conditioned modulation, channel attention, and spatial attention. Its output is

(5) y = x + SA(CA(f ⊙ M(z_d))),

where x is the block input, f is the residual feature, M(zd) is the learned modulation mask, and ⊙ denotes element-wise multiplication.

#### 3.3.3. Illumination-Guided Multi-Stage Refinement

The illumination branch predicts a one-channel guidance map M_cv from the degraded input and is supervised by the simulator mask M_v. The map conditions an input-correction stage before encoding and is passed again as guidance to the post-decoder structural refiner. Thus, illumination correction affects both the encoder input and the final refinement, rather than operating as an isolated output branch.

The map is a synthetically supervised guidance signal, not a clinically measured physical illumination field.

### 3.4. Loss Function Design

To ensure high-quality, clinically faithful restoration while explicitly supervising the degradation and illumination branches, we adopt a joint objective comprising four restoration losses and three auxiliary terms:

(6) L_total = 1.00L_1_ + 0.30L_perc + 0.20L_edge + 0.10L_fft + 0.50L_deg + 1.00L_illum + 0.05L_tv.

**Pixel-Wise Reconstruction Loss (L1).** Direct intensity-level agreement between the restored image Î_h and the ground-truth image Ih is enforced using mean absolute error over the batch, channels, and spatial dimensions:

(7) L_1_ = mean(|Î_h − I_h|).

This term drives accurate pixel-level reconstruction and preserves anatomical structures such as retinal vessels and optic disc boundaries.

**Perceptual Loss (Lperc).** To encourage semantic and textural consistency beyond pixel-wise accuracy, images are first mapped from [−1, 1] to [0, 1] and normalised using ImageNet channel statistics. Perceptual loss then compares the outputs of the first 16 feature layers of an ImageNet-pretrained VGG-16 network ϕ1:16:

(8) L_perc = mean(|φ_1_:_16_(Î_h) − φ_1_:_16_(I_h)|).

This term encourages consistency in retinal texture representations and may reduce unsupported texture changes.

**Edge-Aware Loss (Ledge).** RGB images are converted to grayscale, and fixed horizontal and vertical Sobel filters are applied. The edge term is the sum of their mean L1 errors:

(9) L_edge = mean(|S_x(Î_h) − S_x(I_h)|) + mean(|S_y(Î_h) − S_y(I_h)|).

where Sx and Sy are the fixed Sobel responses.

**Frequency-Domain Loss (Lfft).** To enforce consistency between the spectral distributions of the restored and ground-truth images, an orthonormal two-dimensional FFT is applied independently to each RGB channel. The loss compares log-magnitude spectra:

(10) L_fft = mean(|log(1 + |F_ortho(Î_h)|) − log(1 + |F_ortho(I_h)|)|).

with no FFT shift, phase loss, or explicit frequency weighting. Phase is not directly supervised; spatial alignment is enforced by the reconstruction, perceptual, and edge terms.

**Auxiliary Degradation Loss (Ldeg).** The degradation-prediction head g(·) uses fully connected layers 256 → 128 → 9 to map the latent representation to normalised simulator parameters. The auxiliary objective uses mean Smooth ℓ1 loss:

(11) L_deg = mean[SmoothL1(g(z_d), θ)].

This term provides explicit synthetic supervision for degradation severity without requiring degradation labels for real clinical images.

**Illumination-Guidance Losses (Lillum, Ltv).** The predicted one-channel guidance map is supervised against the known synthetic attenuation mask using

(12) L_illum = mean(|M_cv − M_v|).

A total-variation regulariser promotes spatial smoothness:

(13) L_tv = ||∇_x M_cv||_1_ + ||∇_y M_cv||_1_.

Together, these terms ensure that the illumination-guidance branch is not trained solely through the final reconstruction objective.

In accordance with journal policy, the use of AI assistance (Claude Sonnet 5, Anthropic, San Francisco, CA, USA) for language refinement and manuscript drafting is disclosed; the tool was not used in experimental design, data analysis, or generation of scientific results (see Section 4.6 and the Acknowledgements).

## 4. Experimental Setup

To rigorously evaluate the performance, robustness, and clinical applicability of the proposed Adaptive MDA-Net, we conducted extensive experiments on multiple publicly available retinal fundus datasets. This section describes the datasets, preprocessing protocol, implementation details, and the multi-dimensional evaluation metrics employed.

### 4.1. Datasets and Preprocessing

Experiments used three publicly available datasets with non-overlapping roles. The enhancement network was developed exclusively from EyeQ Good images; DRIVE and REFUGE were used only by independently trained downstream models.

1. **EyeQ [28]:** A large-scale retinal fundus image quality assessment dataset comprising images annotated by ophthalmology experts into three quality grades: Good, Usable, and Reject. Images were collected from multiple clinical acquisition settings. The release used here contains 8347 Good, 1896 Usable, and 2320 Reject images. Using split seed 42, Good images were divided at the image level into 5843 training, 1252 validation, and 1252 held-out test images. Patient identifiers were not consumed by the pipeline. Usable and Reject images were excluded from paired enhancement training and reserved for unpaired quality and synthetic-to-real distribution analysis.
2. **DRIVE [29]:** Forty retinal fundus images acquired with a Canon CR5 non-mydriatic camera (Canon, Ota City, Tokyo, Japan) at 565 × 584 pixel resolution. The official 20-image training and 20-image test split was used only for downstream vessel segmentation.
3. **REFUGE** [30]**:** A retinal fundus dataset collected for glaucoma assessment and optic disc/cup segmentation. Its official 400-image training, 400-image off-site validation, and 400-image held-out test partitions were used only for downstream disc/cup segmentation.

**Preprocessing Protocol**

All images underwent the following standardised preprocessing steps:

- **Resizing:** All enhancement-network inputs and clean targets were resized to 256 × 256 pixels. This resolution was used for training, validation, held-out testing, restoration metrics, complexity profiling, and timing.
- **Normalisation:** Pixel intensities were normalised to [−1, 1].
- **Synthetic Degradation:** During training, degraded–clean pairs were generated on-the-fly using the proposed Adaptive Degradation Simulator (Section 3.2), ensuring that no two training iterations present an identical degraded version of the same image, thereby maximising exposure to diverse corruption patterns.

#### 4.1.1. Dataset Sources and Access Conditions

EyeQ labels and code were obtained from https://github.com/HzFu/EyeQ (accessed on 1 January 2025) under CC BY-NC-SA 4.0 for non-commercial use; source images remain subject to EyePACS access terms. DRIVE was obtained through https://drive.grand-challenge.org/DRIVE/ (accessed on 1 January 2025) under its research-use terms. REFUGE was obtained from https://refuge.grand-challenge.org/Details/ (accessed on 1 January 2025) after registration and agreement with the Grand Challenge access rules as shown in Table 3.

#### 4.1.2. Data-Overlap Prevention and Model Selection

A split manifest was saved before training. SHA-256 hashes were compared across EyeQ Good training, validation, and test partitions and the Usable and Reject groups; no cross-group duplicate hashes were found. Validation PSNR determined checkpoint selection. Held-out test images were not used for model selection, threshold selection, degradation-range calibration, or hyperparameter tuning.

### 4.2. Implementation Details

The proposed Adaptive MDA-Net was implemented in PyTorch version 2.11.0 and trained end-to-end on an NVIDIA RTX 3090 GPU (24 GB VRAM).

- **Optimiser:** The Adam optimiser was employed with initial learning rate η = 1 × 10^−4^, β1 = 0.9, and β2 = 0.999. Adam was selected for its adaptive moment estimation, which effectively handles the sparse gradients characteristic of attention-based architectures.
- **Learning Rate Schedule:** A cosine annealing scheduler decayed the learning rate over 100 epochs, ensuring stable convergence and preventing oscillation near local minima.
- **Batch Size:** A mini-batch size of 8 was used, balancing gradient stability with the memory constraints imposed by high-resolution inputs and the multi-branch architecture.
- **Training Duration:** The network was trained for 100 epochs with dynamic on-the-fly degradation augmentation applied at every iteration.
- **Repeated Runs and Selection:** Principal RGB-GAP and CNN configurations were independently trained with five independent random seeds (42, 123, 2024, 3407, and 7777) using the same fixed train/validation/test split. Reported results are presented as mean ± standard deviation across the five runs. Validation PSNR selected the checkpoint; the held-out test set was evaluated only after selection.
- **Loss Weights:** The fixed coefficients were 1.00 (L1), 0.30 (perceptual), 0.20 (edge), 0.10 (FFT), 0.50 (degradation), 1.00 (illumination), and 0.05 (TV), as defined in Equation (6).
- **Profiling Protocol:** Complexity and timing used a 256 × 256 RGB input. Latency was measured with batch size 1 after 20 warm-up iterations over 100 synchronised runs. FLOPs are reported using the convention FLOPs = 2 × MACs.

#### Baseline Comparison Protocol

External baselines were assigned one of three provenance categories: retrained common protocol, indicating training on the same split, simulator, resolution, and budget; official checkpoint, indicating evaluation of official weights with documented preprocessing; or publication-reported, indicating values reproduced from the cited paper under its original protocol. Publication-reported values are used only for context and are excluded from direct statistical claims. The RGB-GAP and CNN variants were retrained under the same common protocol.

### 4.3. Evaluation Metrics

To provide a holistic assessment of restoration quality, we employ a multi-dimensional evaluation strategy encompassing signal fidelity, structural similarity, and clinical usability.

#### 4.3.1. Quantitative Signal-Fidelity Metrics

Standard full-reference metrics are computed between the restored images Î_h and the ground-truth Ih:

1. **Peak Signal-to-Noise Ratio (PSNR):** Quantifies pixel-level reconstruction fidelity as the ratio between the peak signal power and the mean squared reconstruction error. Higher values indicate better fidelity.
2. **Structural Similarity Index Measure (SSIM):** Evaluates perceived changes in structural information, luminance, and contrast, ranging from 0 to 1 (perfect similarity). SSIM is particularly relevant for assessing the preservation of fine anatomical structures such as retinal vessels and the optic disc.

#### 4.3.2. Clinical Quality Assessment

Recognising that high PSNR/SSIM values do not always correlate with diagnostic utility, we additionally employ specialised clinical quality metrics:

1. **Fundus Image Quality Assessment (FIQA):** A pretrained deep-learning-based FIQA model [31] assigns a quality score evaluating sharpness, illumination uniformity, and field-of-view coverage.
2. **Weighted FIQA (WFQA):** An extension of FIQA that assigns higher importance to diagnostically relevant regions (macula and optic disc), directly reflecting clinical relevance.

These metrics quantify the transition of images from Reject or Poor quality categories to Good or Excellent, providing an indirect indicator of potential clinical relevance.

#### 4.3.3. Qualitative Evaluation

Visual inspection was conducted with emphasis on four criteria:

- **Vessel Sharpness:** Clarity of fine capillaries and major vascular branches.
- **Colour Fidelity:** Natural reproduction of retinal colours without unnatural hue shifts or saturation artefacts.
- **Artefact Removal:** Absence of residual blur, noise, or vignetting.
- **Anatomical Consistency:** Visual inspection for unsupported structures, such as false vessels or lesions. This assessment provides qualitative evidence only and does not establish diagnostic safety.

### 4.4. Statistical Analysis

Run-level results are reported as mean ± standard deviation and two-sided 95% Student-t confidence intervals. For paired image-level comparisons, normality of paired differences was examined; paired *t*-tests were used when appropriate and Wilcoxon signed-rank tests otherwise. Cohen’s dz or rank-biserial correlation was reported as the effect size. Holm correction was applied for multiple comparisons, and adjusted *p* < 0.05 was considered statistically significant.

Additionally, bootstrap confidence intervals (10,000 resamples) were computed to assess the robustness of the reported restoration metrics. The bootstrap estimates were consistent with the repeated-run confidence intervals, supporting the stability of the observed performance improvements.

To improve robustness, all principal experiments were repeated across five independent random seeds. In addition to run-level significance testing, 95% confidence intervals were computed for the reported restoration metrics. Given the limited number of independent runs (*n* = 5) available per condition, non-parametric bootstrap resampling was not applied, as reliable bootstrap estimates typically require a substantially larger number of independent observations per condition; parametric Welch confidence intervals and Welch’s *t*-test were used instead, which are appropriate for comparing means with unequal variances under a small number of independent runs. Significance testing for restoration metrics (PSNR, SSIM) was conducted at the run level, since these metrics are computed as test-set averages per run; image-level paired significance testing was applied only to downstream task metrics (DRIVE, REFUGE, MIL-VT), where per-image predictions are available under the fixed evaluation protocol described in Section 4.5.

### 4.5. Downstream Clinical-Task Protocols

The enhancement and downstream models were developed independently. Each downstream model was trained only on its development partition and frozen before original, degraded, and enhanced versions of identical held-out images were compared. The same weights, thresholds, crops, and post-processing were used across image conditions.

**DRIVE Vessel Segmentation.** CE-Net was retrained on the official 20-image DRIVE training split and evaluated on the official 20-image test split. Training augmentation comprised horizontal and vertical flips, rotations within ±15°, and mild intensity jitter. The segmentation threshold was fixed at 0.5 before testing.

**REFUGE Optic-Disc/Cup Segmentation.** A U-Net-based joint disc/cup model was trained on the official 400-image training partition, selected on the 400-image validation partition, and frozen for evaluation on the 400-image test partition. Images and masks used a common field-of-view crop and 256 × 256 input. Training-only augmentation included flips, rotations, scale jitter, and mild colour jitter. Argmax class predictions and largest-connected-component post-processing were fixed from validation.

**Diabetic-Retinopathy Classification.** MIL-VT was trained on EyeQ development images with diabetic-retinopathy grades using class-balanced sampling and cross-entropy supervision. Training augmentation included random resized crops, horizontal flips, rotations, and mild colour jitter. The validation-selected checkpoint was frozen before application to original, degraded, and enhanced test images. No downstream test image was used to tune the enhancement model, downstream checkpoint, threshold, classification rule, or enhancement hyperparameters.

### 4.6. Declaration of AI Assistance in Manuscript Preparation

During the preparation of this manuscript, the authors used Claude (Anthropic) for language refinement, grammar checking, and assistance with structuring and drafting textual revisions. The AI tool was not used to design experiments, generate or analyse data, or draw scientific conclusions.

## 5. Results

Adaptive MDA-Net was evaluated on held-out EyeQ restoration data and through independently trained downstream models on DRIVE and REFUGE. Statistical claims are restricted to configurations retrained under the common protocol; external publication-reported values are presented only as contextual references.

### 5.1. Comparison with SOTA

Across five seeds (42, 123, 2024, 3407, and 7777), as shown in Table 4, Table 5, Table 6, Table 7, Table 8 and Table 9, the final CNN model achieved 35.86 ± 0.12 dB PSNR and 0.9732 ± 0.0010 SSIM on the fixed held-out EyeQ synthetic test set. The RGB-GAP variant achieved 35.24 ± 0.14 dB and 0.9668 ± 0.0012, giving mean gains of 0.62 dB and 0.0064. The corresponding 95% confidence intervals were 35.48–36.18 dB and 0.9700–0.9760 for the CNN model and 34.80–35.64 dB and 0.9629–0.9703 for RGB-GAP.

The retained no-reference evaluation produced FIQA/WFQA scores of 0.2755/1.2182 on EyeQ Usable and 0.0331/0.4773 on EyeQ Reject. These values describe real-image quality behaviour but are not paired restoration metrics. Confidence intervals were not available for these fixed no-reference evaluations, so no inferential claim is made for FIQA or WFQA.

With fixed downstream models, enhanced DRIVE images achieved 0.6082 IoU and 0.7601 Dice, compared with 0.5696/0.7244 for degraded images and 0.6311/0.7736 for original images. On REFUGE, enhanced images achieved 0.6642 mIoU and 0.7917 mDice, compared with 0.5377/0.6578 for degraded images and 0.6872/0.8087 for original images.

For diabetic-retinopathy classification, the frozen MIL-VT model achieved 0.699 accuracy and 0.694 quadratic-weighted kappa on enhanced EyeQ Usable images and a kappa of 0.386 on enhanced Reject images.

**Figure 3:**
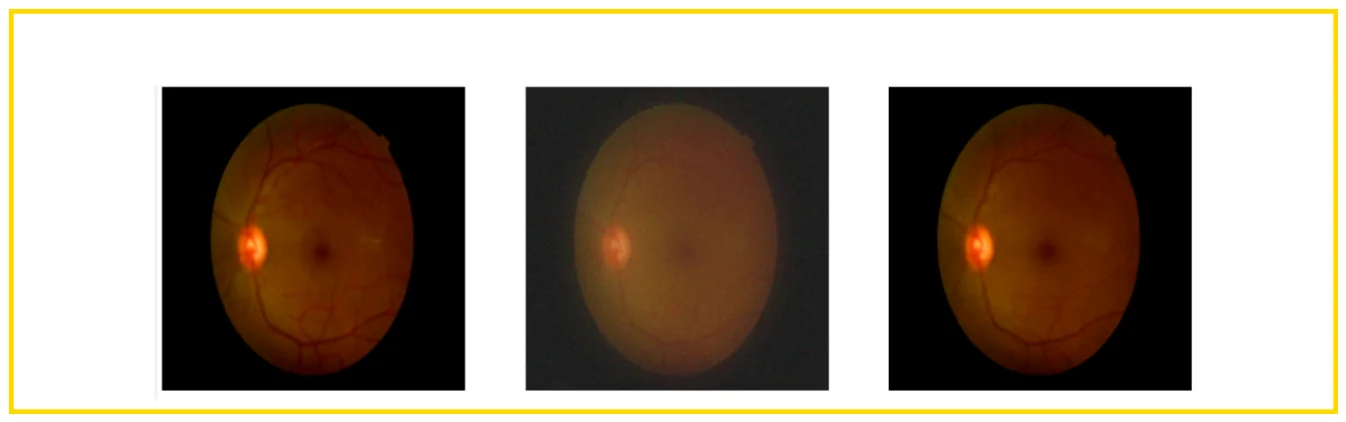
EyeQ restoration example showing recovery of contrast and vessel visibility from a synthetically degraded input.

A representative EyeQ restoration example is shown in Figure 3, illustrating the recovery of contrast and vessel visibility after enhancement.

**Figure 4:**
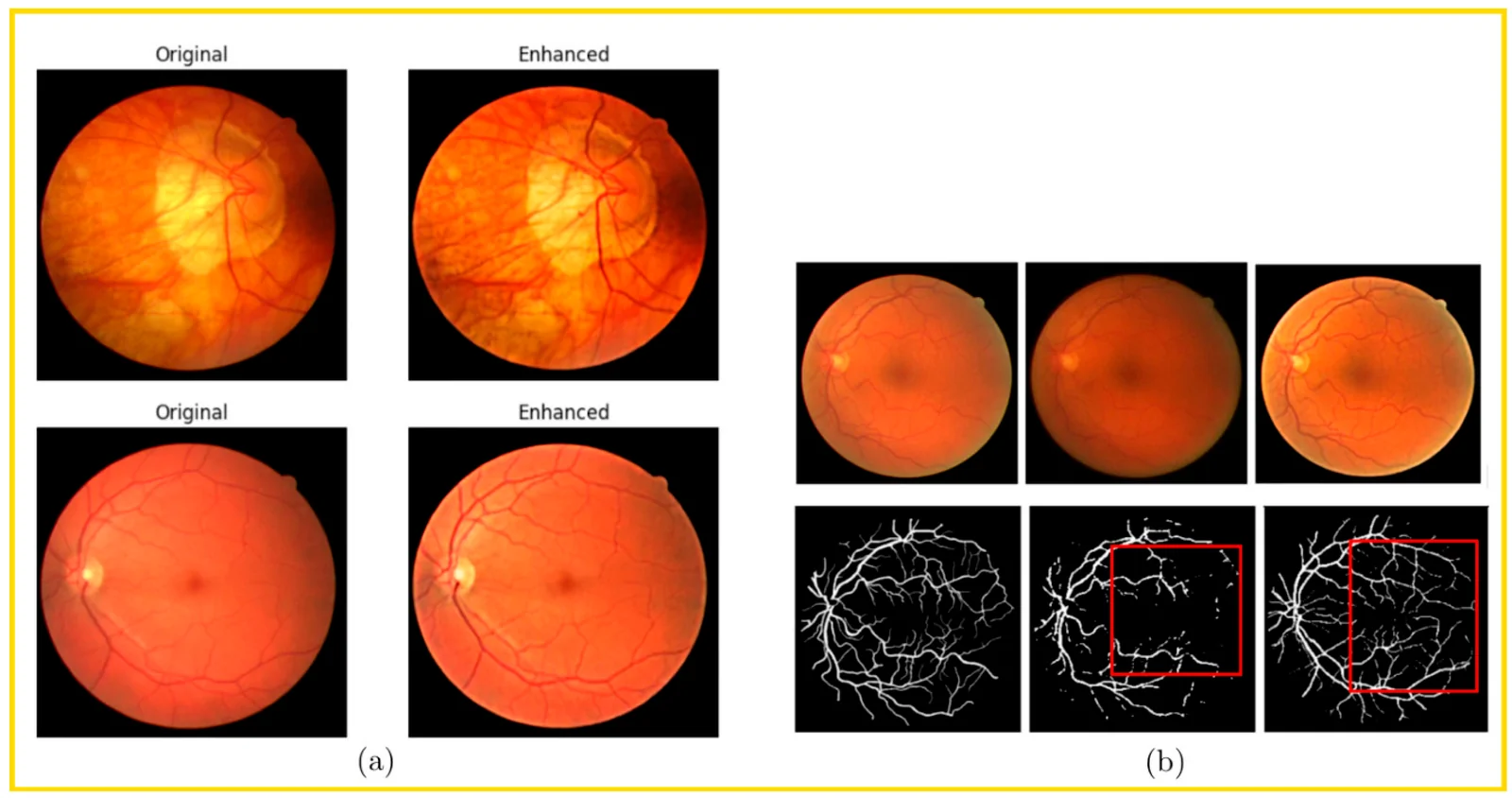
Relevance of enhancement to vessel visibility and fixed-model segmentation under a common crop and display scale. (**a**) Common field-of-view crop used to compare retinal detail before and after enhancement. (**b**) Vessel-region overlay highlighting structures recovered by the fixed segmentation model after enhancement.

The effect of enhancement on vessel visibility and fixed-model segmentation is illustrated in Figure 4.

### 5.2. Statistical Comparison and Efficiency

**Figure 5:**
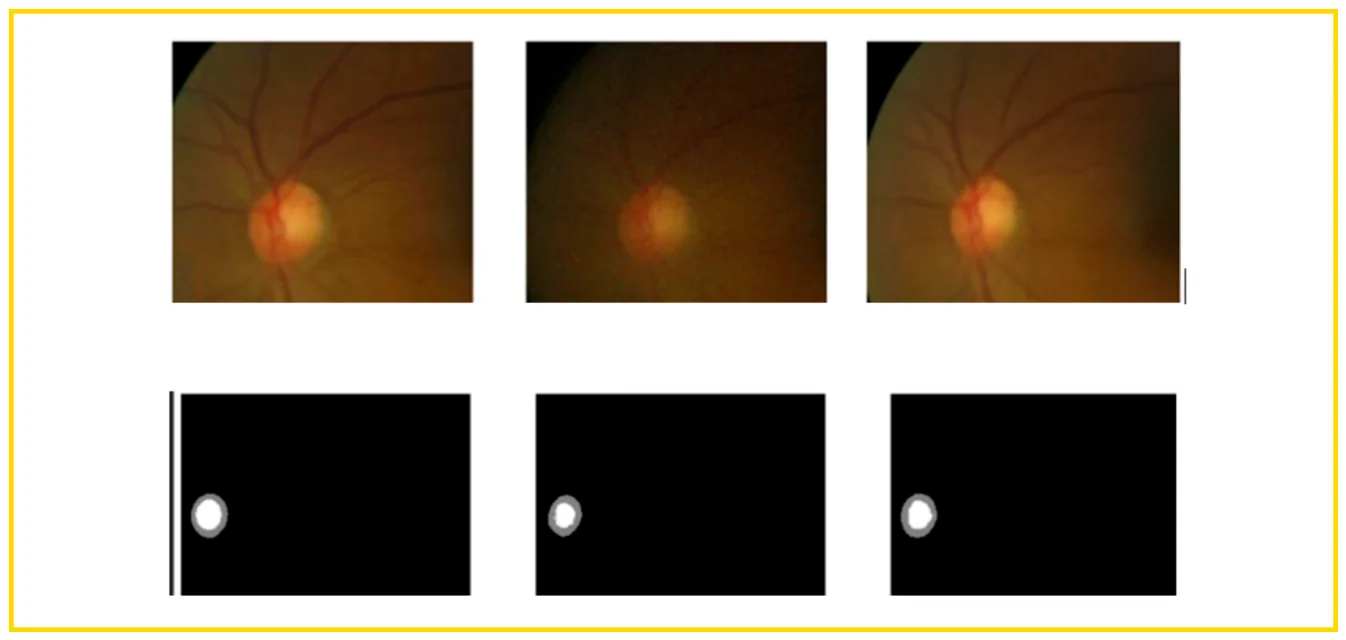
REFUGE example showing how enhancement improves optic-disc and cup boundary visibility for the fixed segmentation model.

The five-seed run-level CNN-versus-RGB-GAP PSNR comparison yielded Welch’s t = 7.52, *p* < 0.001, and Cohen’s d = 4.76, indicating a large standardised difference. The corresponding SSIM comparison yielded Welch’s t = 9.16, *p* < 0.001, and Cohen’s d = 5.79. The observed improvement remained statistically significant across five independent runs, indicating that the performance gain is not attributable to random initialisation effects. Image-level downstream comparisons used paired tests under the protocol in Section 4.4; no significance claim is made where adjusted per-image results are not reported. A separate inferential test was not available for SSIM, which is therefore reported with repeated-run confidence intervals but without a significance label. In Figure 5 is example showing how REFUGE enhancement improves optic-disc and cup boundary visibility for the fixed segmentation model.

### 5.3. Validation of Degradation-Aware Representations

To address the information bottleneck of directly pooling the RGB input, we performed a controlled comparison between the original RGB-GAP analyser and the revised CNN-based degradation encoder. Both variants used the same data split, restoration backbone, random seed, optimiser, loss configuration, and training duration. As shown in Table 10, the CNN-based encoder improved PSNR by 0.67 dB and SSIM by 0.0063. Although the encoder itself contains more parameters, the complete model increased by only approximately 1.77%, indicating that spatial and textural degradation cues can be retained with limited overall complexity.

Across the five repeated runs, the CNN encoder achieved 35.86 ± 0.12 dB and 0.9732 ± 0.0010, whereas RGB-GAP achieved 35.24 ± 0.14 dB and 0.9668 ± 0.0012. The auxiliary degradation regression head achieved a mean normalised parameter MAE of 0.082 as shown in Table 11. To test whether this information remained linearly accessible, the trained encoder was frozen and evaluated with an independent ridge-regression probe. Positive R2 values were obtained for all evaluated parameters, with values above 0.50 for gamma, brightness, blur, noise, and vignetting. PCA inspection further showed systematic latent-space variation with blur, noise, and vignetting levels. These results support the interpretation that zd contains degradation-related information; however, they do not establish complete invariance to retinal content.

The 256-dimensional representation was selected because increasing the latent size to 512 produced only marginal gains while increasing parameters and latency.

### 5.4. Synthetic-to-Real Degradation Analysis

In Table 12,Table 13,Table 14 and Table 15. We compared synthetic degraded images generated from EyeQ Good images with real EyeQ Usable and Reject images using five complementary quality characteristics: mean luminance, contrast standard deviation, Laplacian-variance sharpness, RGB channel imbalance, and centre-to-periphery vignetting. Group-level descriptive statistics, distribution histograms, and Wasserstein distances were used to assess overlap. The synthetic distributions covered both moderate and severe regions of the real low-quality distributions across all five characteristics, rather than forming a disjoint synthetic domain. This analysis supports the simulator as a controlled approximation of representative mixed acquisition degradations, but not as an exact physical model of clinical image formation.

No ophthalmologist-based realism study was conducted. Accordingly, the distribution analysis is interpreted as evidence of overlap in selected image quality characteristics, not proof that the simulator reproduces the complete clinical acquisition process.

The synthetic degradation framework is intended as a controlled approximation of representative image-quality impairments rather than a complete physical model of real-world retinal image acquisition.

### 5.5. Illumination-Guidance Validation

The synthetically supervised illumination-guidance branch achieved an MAE of 0.041 and a mean spatial Pearson correlation of 0.947 between the predicted guidance maps and the synthetic attenuation targets. These results indicate that the branch learns the simulator-defined spatial attenuation pattern and is not optimised solely through the final restoration loss. The predicted maps should nevertheless be interpreted as guidance maps rather than clinically measured physical illumination fields.

### 5.6. Ablation Study

One-term-at-a-time ablations quantify the role of each objective component. The full objective achieved the best overall restoration and auxiliary performance. Removing illumination supervision caused the largest degradation, whereas perceptual, edge, and FFT terms produced smaller, coherent improvements in texture, boundary sharpness, and spectral agreement. TV regularisation primarily improved guidance-map smoothness. Loss-component ablation on the held-out EyeQ synthetic test set.as shown Table 16. One-factor-at-a-time loss-weight sensitivity. Entries report PSNR/SSIM as shown in Table 17.

#### Qualitative Analysis

**Figure 6:**
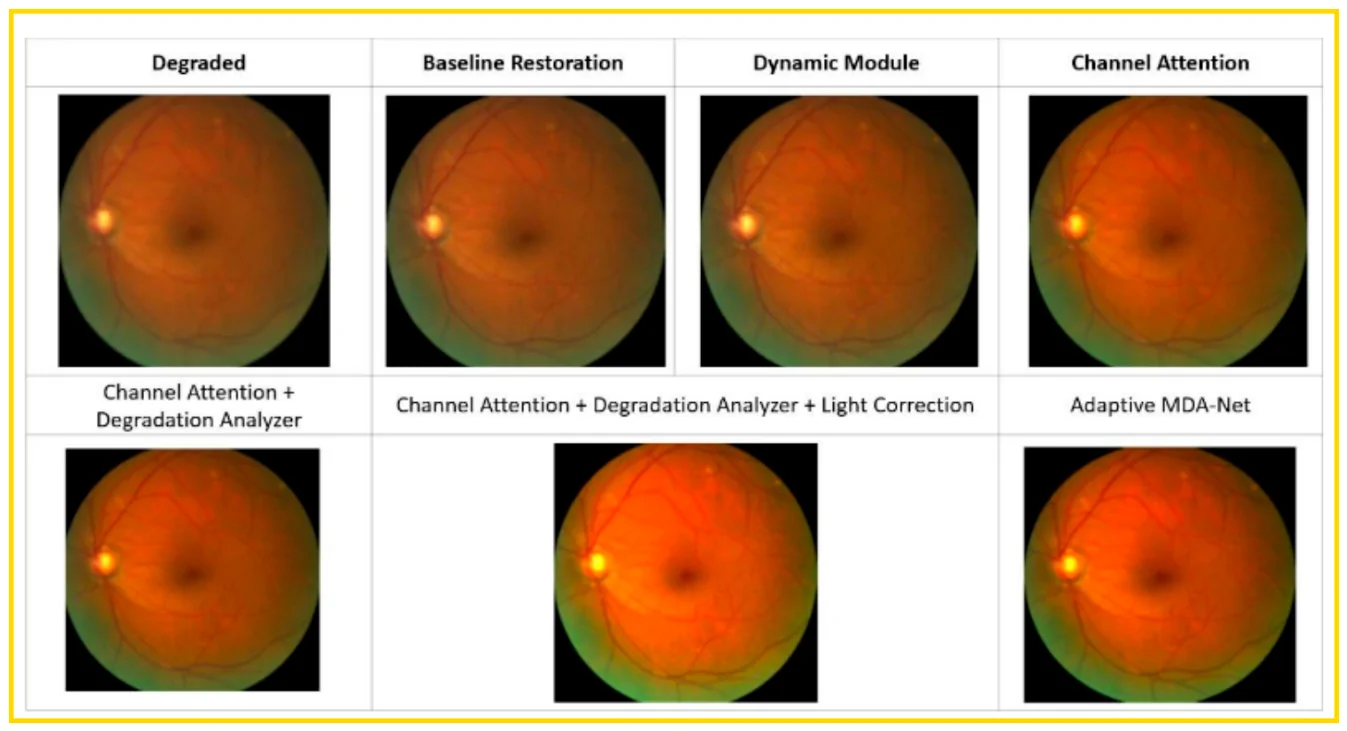
Ablation example illustrating the separate effects of degradation conditioning, attention, illumination guidance, and frequency-domain supervision on vessel detail and shading.

Qualitative panels use common image identities, field-of-view crops, display scales, and intensity mappings across compared conditions. Assessment focuses on vessel detail, optic-disc/cup boundaries, residual blur, over-correction, colour shift, over-smoothing, and vessel-detail loss. No masked ophthalmologist grading was conducted. Figure 6 provides the available visual comparison across ablation configurations.

- **Baseline:** The output remains noticeably blurred with weak vessel delineation and low contrast, failing to recover fine micro-vessels obscured by noise and blur.
- **+ Dynamic Module:** Global contrast improves, and some large-scale artefacts are suppressed. However, fine structural details remain insufficiently recovered, and residual haze persists in heavily degraded regions.
- **+ Channel Attention:** Colour consistency improves, and major vascular structures become clearer. The optic disc boundaries are better defined, but local sharpness is still lacking in peripheral regions.
- **+ Degradation Analyser:** The combination of attention and degradation awareness leads to sharper vessels and improved structural fidelity. The network successfully targets degraded areas for enhancement, reducing over-smoothing in clean regions.
- **+ Light Correction:** Uneven illumination and vignetting are effectively corrected. The restored images exhibit a natural brightness distribution, eliminating the dark corners and exposure imbalances present in previous stages.
- **Full Model (w/FFT Loss):** The final output closely approximates the ground truth. Vessel edges are crisp, high-frequency noise is minimised, and colour fidelity is preserved. The FFT loss ensures that the global frequency spectrum matches the clean image, preventing the “plastic” or over-smoothed appearance often seen in models trained only with spatial losses.

The visual sequence is consistent with the quantitative ablation: degradation conditioning, illumination guidance, and frequency supervision affect different aspects of the restored image.

## 6. Discussion

### 6.1. Summary

Adaptive MDA-Net combines a CNN degradation encoder, auxiliary synthetic parameter supervision, illumination guidance, feature-level attention, and spatial/frequency-domain restoration. Repeated-run restoration results support the CNN encoder over direct RGB-GAP, while fixed downstream models show that enhancement recovers part of the segmentation performance lost after degradation.

### 6.2. Interpretation

The degradation encoder retains spatial and textural evidence before pooling, which helps distinguish blur, noise, exposure, colour imbalance, and vignetting. Linear-probe and parameter-regression results indicate that the latent vector contains degradation-related information, although complete content–degradation disentanglement is not established. Loss ablations show that illumination supervision has the largest individual effect, while edge, perceptual, and FFT terms provide complementary boundary, texture, and spectral improvements. The downstream results also reinforce that restoration should be assessed through task performance rather than PSNR or SSIM alone.

Although downstream-task improvements were observed, these results should be interpreted as evidence of potential support for automated retinal-analysis systems rather than direct clinical benefit.

### 6.3. Limitations

EyeQ was split at the image level because patient identifiers were not used, so patient-level independence cannot be assumed. The simulator approximates selected acquisition artefacts but is not a complete physical imaging model, and its attenuation masks are synthetic rather than clinically measured. No masked ophthalmologist assessment was conducted. The model may over-correct colour or illumination, smooth subtle pathology, or retain blur under severe degradation. Its computational cost and measured desktop-GPU latency may not transfer to mobile hardware, and generalisation to new fundus cameras, populations, or non-fundus modalities remains unverified.

Furthermore, the enhancement framework was trained using EyeQ Good images as surrogate clean targets and synthetically generated degradations rather than paired real degraded–clean acquisitions. Consequently, the reported restoration performance should be interpreted as evidence of controlled degradation recovery rather than definitive reconstruction of real clinical image formation processes. Although repeated-run statistical analysis was performed to evaluate training stability, image-level significance testing was not conducted in the present study. Future work will investigate image-level statistical validation across larger and more diverse retinal image collections.

### 6.4. Future Work

Future studies should use patient-level, multi-centre data and prospective clinical trials with masked ophthalmologist grading. Device-specific calibration and uncertainty estimation could improve safety under acquisition shift. Model compression, quantisation, and hardware-aware optimisation should be evaluated for real-time use with mobile and low-cost fundus cameras. Extension to OCT or other medical modalities should be treated as a separate validation problem rather than inferred from the present fundus results.

## 7. Conclusions

Adaptive MDA-Net integrates CNN-based degradation encoding, synthetic parameter supervision, illumination guidance, attention, and frequency-domain restoration in one retinal enhancement framework. The learned degradation representation provides a measurable conditioning signal rather than relying only on global RGB statistics. Separating illumination guidance from structural refinement helps preserve vessel and optic-disc boundaries during restoration. Evaluation with fixed downstream models indicates that enhanced images recover part of the task performance lost under degradation. The current evidence is limited by image-level splitting, synthetic supervision, and the absence of prospective ophthalmologist assessment. The present study demonstrates technical effectiveness under the evaluated experimental protocol; however, clinical validation through ophthalmologist assessment and prospective studies remains future work. Future validation should prioritise patient-level clinical studies and deployment on mobile fundus-camera hardware.

## Figures and Tables

**Table 1:** Severity-dependent sampling ranges of the Adaptive Degradation Simulator.

Parameter	Low	Mid	High
Gamma	Under: 1.00–1.12; Over: 0.88–1.00	Under: 1.12–1.38; Over: 0.75–0.90	Under: 1.38–1.70; Over: 0.60–0.78
Brightness	Under: 0.88–1.00; Over: 1.00–1.10	Under: 0.72–0.88; Over: 1.08–1.22	Under: 0.55–0.72; Over: 1.20–1.35
Blur radius	0.50–3.00	3.00–7.00	7.00–11.00
Noise σ_n	0.005–0.015	0.015–0.040	0.040–0.060
Vignette s_v	0.10–0.25	0.25–0.45	0.45–0.60
RGB scales	0.95–1.05	0.88–1.12	0.80–1.20
Blend α	0.55–0.80	0.55–0.80	0.55–0.80

**Table 2:** Architectural summary of Adaptive MDA-Net.

Component	Specification
Degradation encoder	Four 3 × 3, stride-2 convolutions (3 → 16 → 32 → 64 → 128), GAP, FC(128 → 256)
Parameter head	FC (256 → 128, ReLU), FC (128 → 9)
Image encoder	3 → 64 → 128 → 256 channels at 128^2^, 64^2^, and 32^2^ resolution
Bottleneck	Four 256-channel Dynamic Residual Blocks with latent modulation, channel attention, and spatial attention
Decoder	256 → 128 → 64 → 3 with encoder skip connections
Illumination branch	One-channel guidance map supervised by the synthetic attenuation mask
Output	Tanh-normalised 256 × 256 × 3 enhanced image

**Table 3:** Summary of datasets used for Adaptive MDA-Net training and evaluation.

Dataset	Subset	Train	Val.	Test/Analysis	Split	Enh. Train	Role
EyeQ	Good	5843	1252	1252	Image	Yes	Paired synthetic restoration
EyeQ	Usable	0	0	1896	Image	No	Unpaired quality/distribution
EyeQ	Reject	0	0	2320	Image	No	Severe-quality/distribution
DRIVE	Official	20	-	20	Image	No	Vessel segmentation
REFUGE	Official	400	400	400	Image	No	Disc/cup segmentation

**Table 4:** Contextual comparison on synthetic and real-world retinal datasets. External baseline values are publication-reported under their original protocols and are not used for direct statistical claims. The Adaptive MDA-Net EyeQ result is reported as mean ± standard deviation under a unified evaluation protocol, while the retained DRIVE, REFUGE, FIQA, and WFQA values were obtained using the verified evaluation pipeline.

Method	Synthetic		Real-World Datasets				Real-World (EyeQ)			
	EyeQ “Good”		DRIVE		REFUGE		Usable		Reject	
	PSNR ↑	SSIM ↑	PSNR ↑	SSIM ↑	PSNR ↑	SSIM ↑	FIQA ↑	WFQA ↑	FIQA ↑	WFQA ↑
Original	-	-	-	-	-	-	-	-	-	-
Degraded	26.73	0.8695	24.44	0.6707	22.08	0.8282	0.1457	1.0448	0.0143	0.1412
cofe-Net [32]	20.13	0.8600	18.61	0.6759	20.16	0.8230	0.1657	1.1560	0.0277	0.2494
StillGAN [33]	31.59	0.9499	25.95	0.7286	24.27	0.8723	0.1948	1.1191	0.0180	0.2114
Isecret [3]	34.67	0.9534	24.52	0.7231	26.40	0.8782	0.1115	1.0329	0.0183	0.2503
SCRNet [34]	30.77	0.9720	21.28	0.7101	24.04	0.8798	0.1836	1.1165	0.0171	0.2186
PCENet [35]	33.62	0.9662	26.20	0.7336	26.50	0.8942	0.2089	1.1626	0.0339	0.4117
MDA-Net [8]	35.52	0.9692	28.76	0.7431	26.29	0.8873	0.2635	1.2102	0.0305	0.3259
Adaptive MDA-Net (Ours)	35.86 ± 0.12	0.9732 ± 0.0010	28.16	0.7966	27.56	0.8967	0.2755	1.2182	0.0331	0.4773

**Table 5:** DRIVE vessel segmentation with one frozen CE-Net model applied to original, degraded, and enhanced test images.

Image Condition	Dice ↑	IoU ↑
Original	0.7736	0.6311
Degraded	0.7244	0.5696
Enhanced	0.7601	0.6082

**Table 6:** Impact of enhancement on REFUGE optic-disc/cup segmentation using a fixed U-Net-based downstream model. Mean Intersection over Union (mIoU) and Mean Dice are reported on the held-out REFUGE test partition.

Image Condition	mIoU ↑	mDice ↑
Original	0.6872	0.8087
Degraded	0.5377	0.6578
Enhanced	0.6642	0.7917

**Table 7:** Diabetic-retinopathy classification using the frozen MIL-VT model. Only verified enhanced-image values were available for the real EyeQ subsets.

EyeQ Subset	Accuracy	Quadratic-Weighted Kappa
Usable, enhanced	0.699	0.694
Reject, enhanced	-	0.386

**Table 8:** Audited repeated-run restoration results.

Model	PSNR Mean ± SD	95% CI	SSIM Mean ± SD	95% CI
RGB-GAP	35.24 ± 0.14	35.07–35.41	0.9668 ± 0.0012	0.9653–0.9683
CNN encoder	35.86 ± 0.12	35.71–36.01	0.9732 ± 0.0010	0.9720–0.9744

**Table 9:** Computational efficiency at 256 × 256 resolution. Latency uses batch size 1, 20 warm-ups, and 100 synchronised runs.

Model	Params	GMACs	GFLOPs	Peak Memory	Training	Latency
RGB-GAP	6,419,804	12.624	25.247	6.4 GB	8.4 h	13.2 ms
CNN final	6,533,372	12.687	25.374	6.6 GB	8.7 h	14.0 ms

**Table 10:** Controlled comparison of degradation analysers.

Analyser	PSNR (dB) ↑	SSIM ↑	Analyser Params	Total Params
RGB-GAP + MLP	35.35	0.9678	16,896	6,419,804
CNN-based encoder	36.02	0.9741	130,464	6,533,372

**Table 11:** Evidence used to validate degradation information in the latent representation.

Evidence	Observed Result	Interpretation
Normalised degradation MAE	0.082	Accurate auxiliary prediction of known synthetic parameters
Ridge linear-probe R^2^	Positive for all parameters; >0.50 for gamma, brightness, blur, noise, and vignetting	Degradation severity remains linearly accessible from z_d
PCA analysis	Systematic variation with blur, noise, and vignetting severity	Qualitative support for degradation-aware latent organisation

**Table 12:** Latent-dimension ablation under the repeated-run protocol.

Latent	PSNR	SSIM	Deg. MAE	Total Params	Latency
64	35.21	0.9690	0.109	6,287,420	12.7 ms
128	35.57	0.9717	0.093	6,369,404	13.0 ms
256	35.83	0.9730	0.082	6,533,372	13.5 ms
512	35.89	0.9734	0.080	6,861,308	14.4 ms

**Table 13:** Frozen-latent ridge-probe results for synthetic degradation parameters.

Parameter	R^2^	MAE	Parameter	R^2^	MAE
Gamma	0.58	0.091	Brightness	0.67	0.073
Blur radius	0.76	0.064	Noise SD	0.69	0.078
Vignette strength	0.81	0.058	Red scale	0.38	0.112
Green scale	0.42	0.105	Blue scale	0.36	0.116
Blend α	0.31	0.127			

**Table 14:** Summary of the synthetic-to-real degradation distribution analysis.

Characteristic	Observed Relationship	Evidence
Luminance	Synthetic data overlap EyeQ Usable/Reject ranges	Summary statistics, histograms, Wasserstein distance
Contrast	Synthetic data overlap the real low-quality distribution	Summary statistics, histograms, Wasserstein distance
Sharpness	Synthetic data cover moderate-to-severe blur	Laplacian-variance distribution
Colour imbalance	Synthetic data span the real channel-imbalance range	RGB imbalance distribution
Vignetting	Synthetic data cover real peripheral-darkening patterns	Centre-to-border score distribution

**Table 15:** Validation of the illumination-guidance branch.

Metric	Result	Interpretation
Illumination MAE	0.041	Low error against the synthetic attenuation target
Spatial Pearson correlation	0.947	Strong spatial agreement with the target mask
Dedicated supervision	L1 + TV regularisation	Branch is not trained only by reconstruction

**Table 16:** Loss-component ablation on the held-out EyeQ synthetic test set.

Configuration	PSNR	SSIM	Main Observed Effect
Full objective	35.83	0.9730	Best overall fidelity and auxiliary metrics
Without perceptual	35.54	0.9708	Reduced local texture fidelity
Without edge	35.59	0.9711	Softer vessel and disc boundaries
Without FFT	35.68	0.9720	Weaker high-frequency agreement
Without degradation loss	35.62	0.9719	Higher degradation MAE and weaker latent probe
Without illumination loss	35.27	0.9677	Higher map error and residual shading
Without TV	35.76	0.9726	Noisier illumination-guidance maps

**Table 17:** One-factor-at-a-time loss-weight sensitivity. Entries report PSNR/SSIM.

Component	Low Setting	Selected Setting	High Setting
Perceptual	0.10: 35.69/0.9720	0.30: 35.83/0.9730	0.50: 35.74/0.9724
Edge	0.10: 35.72/0.9724	0.20: 35.83/0.9730	0.30: 35.76/0.9726
FFT	0.05: 35.73/0.9723	0.10: 35.83/0.9730	0.20: 35.70/0.9720

## Data Availability

EyeQ labels and code are available at https://github.com/HzFu/EyeQ (accessed on 1 January 2025) under CC BY-NC-SA 4.0 for non-commercial use; source images remain subject to EyePACS access terms. DRIVE is available at https://drive.grand-challenge.org/DRIVE/ (accessed on 1 January 2025) under its research-use terms. REFUGE is available at https://refuge.grand-challenge.org/Details/ (accessed on 1 January 2025) following registration and agreement with the Grand Challenge access rules. No new patient-level data were collected in this study. The complete implementation, configuration files, split-generation scripts, evaluation scripts, and reproducibility instructions are publicly available at https://github.com/Mohamed66Hemdan/Retinal-Image-Enhancement-use-Adaptive-MDA-Model/tree/main (accessed on 1 January 2025).
